# Draft Genome Sequence of Shiga Toxin-Negative *Escherichia coli* O157:H7 Strain C1-057, Isolated from Feedlot Cattle

**DOI:** 10.1128/genomeA.00049-16

**Published:** 2016-03-03

**Authors:** Hua Yang, Brandon Carlson, Ifigenia Geornaras, Dale Woerner, John Sofos, Keith Belk

**Affiliations:** a Department of Animal Sciences, Center for Meat Safety & Quality, Colorado State University, Fort Collins, Colorado, USA; b Cargill Turkey & Cooked Meats, Nebraska City, Nebraska, USA

## Abstract

*Escherichia coli* O157:H7 is one of the major foodborne pathogens in the United States. We isolated a variant Shiga toxin-negative *E. coli* O157:H7 strain from feedlot cattle. We report here the draft genome sequence of this isolate, consisting of a chromosome of ~4.8 Mb and two plasmids of ~96 kb and ~14 kb.

## GENOME ANNOUNCEMENT

*Escherichia coli* serotype O157:H7 is a major foodborne pathogen frequently involved in outbreaks of foodborne disease in the United States (1). One of the classical virulence factors of *E. coli* O157:H7 is the production of Shiga toxins. Persistent uptake of Shiga toxins may ultimately result in the life-threatening hemolytic-uremic syndrome (2). Cattle are considered the major reservoir of *E. coli* O157:H7 (3, 4). Understanding the nature of *E. coli* O157:H7 colonization of feedlot cattle will help with the development of interventions to minimize its entry into harvest facilities, with the purpose of controlling the contamination of end meat products with this pathogen.

*E. coli* O157:H7 strain C1-057 was isolated from a rectal fecal sample grabbed from feedlot cattle. It was characterized by using a five-gene multiplex PCR that detects gene fragments unique to serotype O157:H7 (*rfbE* encoding the O157 antigen and *fliC_h7_* encoding the H7 antigen), as well as three key virulence genes (*eae* encoding intimin, *stx*_1_ gene encoding Shiga toxin 1, and *stx*_2_ encoding Shiga toxin 2). The PCR results confirmed that strain C1-057 belonged to serotype O157:H7, as it carried the *eae* gene; however, it did not carry either *stx*_1_ or *stx*_2_ gene (i.e., was *stx* negative) (5). The genomic characterization of this variant Shiga toxin-negative strain will help to gain insight into its relationship with Shiga toxin-producing *E. coli* O157:H7 isolates commonly carried by feedlot cattle.

Therefore, we sequenced the genome of this Shiga-toxin negative *E. coli* O157:H7 strain. Strain C1-057 was cultured in Trypticase soy broth (TSB) (Becton & Dickinson, NJ) overnight at 37°C. The genomic DNA of strain C1-057 was extracted from pure culture using the E.Z.N.A. bacterial DNA kit (Omega Bio-tek, Norcross, GA). A Pacific Biosciences RSII system (PacBio, University of Washington, Seattle, WA) was used to obtain the complete genome sequences. A 3- to 20-kb library of the strain was prepared and sequenced using C_2_ chemistry kits on one single-molecule real-time (SMRT) cell with a 90-min collection protocol, achieving an average genome coverage of >100× for the strain. The 3- to 20-kb continuous-long-read (CLR) data were *de novo* assembled using the PacBio Hierarchical Genome Assembly Process 2 (HGAP2)/Quiver software package. When fully assembled, the genome consisted of seven scaffolds: scaffolds 1 to 5, representing the complete chromosome of 4,783,867 bp, and scaffolds 6 and 7, representing a 96,420-bp plasmid and a 13,853-bp plasmid, respectively. These sequences were annotated using the NCBI Prokaryotic Genome Annotation Pipeline (PGAP) (http://www.ncbi.nlm.nih.gov/genomes/static/Pipeline.html). Further comparative analysis with other Shiga toxin-positive *E. coli* O157:H7 isolates will provide valuable information to understand the evolution of specific properties related to the colonization and adaptation of *E. coli* O157:H7 strains to feedlot cattle.

### Nucleotide sequence accession numbers.

This whole-genome shotgun project has been deposited at DDBJ/EMBL/GenBank under the accession no. LAZO00000000. The version described in this paper is version LAZO01000000.
